# Supplementary material for: Facile and Efficient Syntheses of (11Z,13Z)-Hexadecadienal and Its Derivatives: Key Sex Pheromone and Attractant Components of Notodontidae
Source: Molecules. 2019 May 8;24(9):1781. doi: 10.3390/molecules24091781 (PMC6540594; doi:10.3390/molecules24091781)
Supplement: Supplementary file 1 [file molecules-24-01781-s001.pdf]

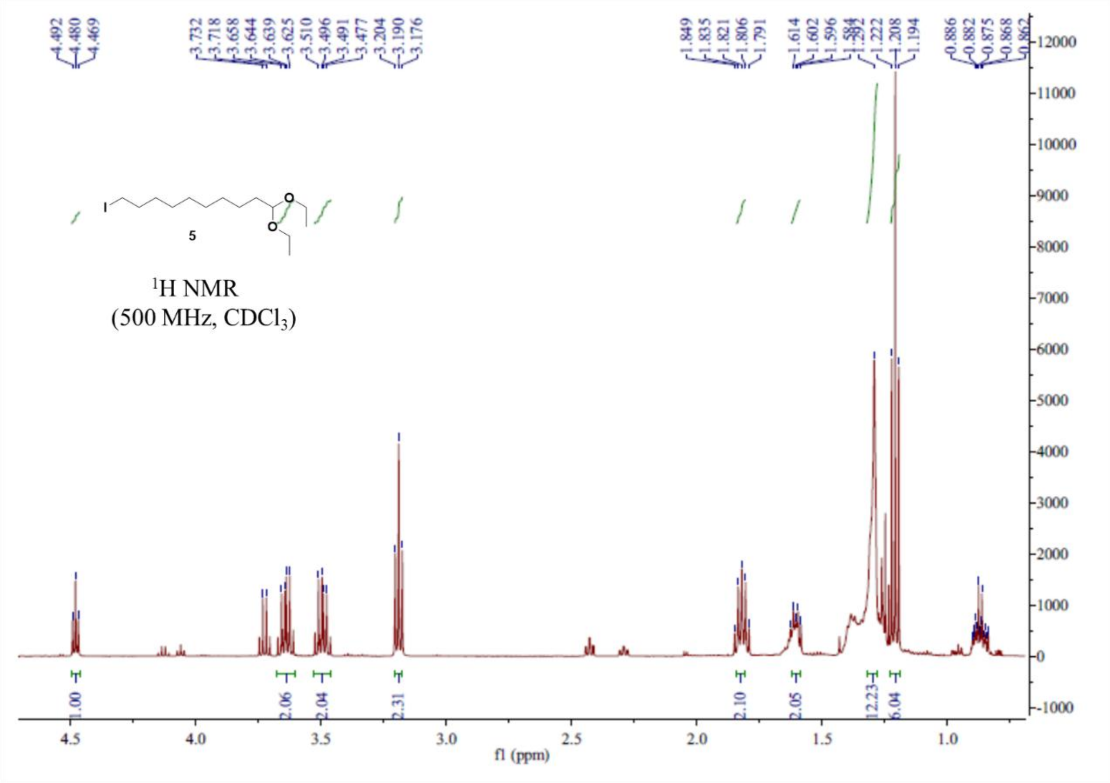

The ^{1}H NMR spectrum of compound **5**

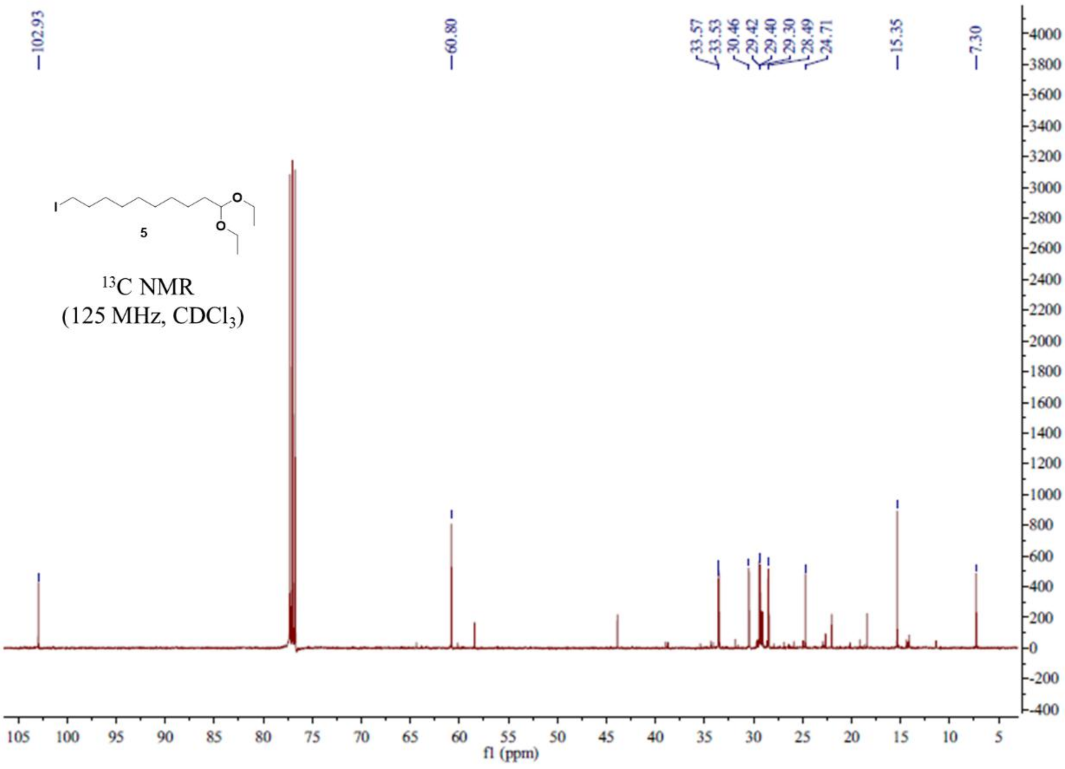

The ^{13}C NMR spectrum of compound **5**

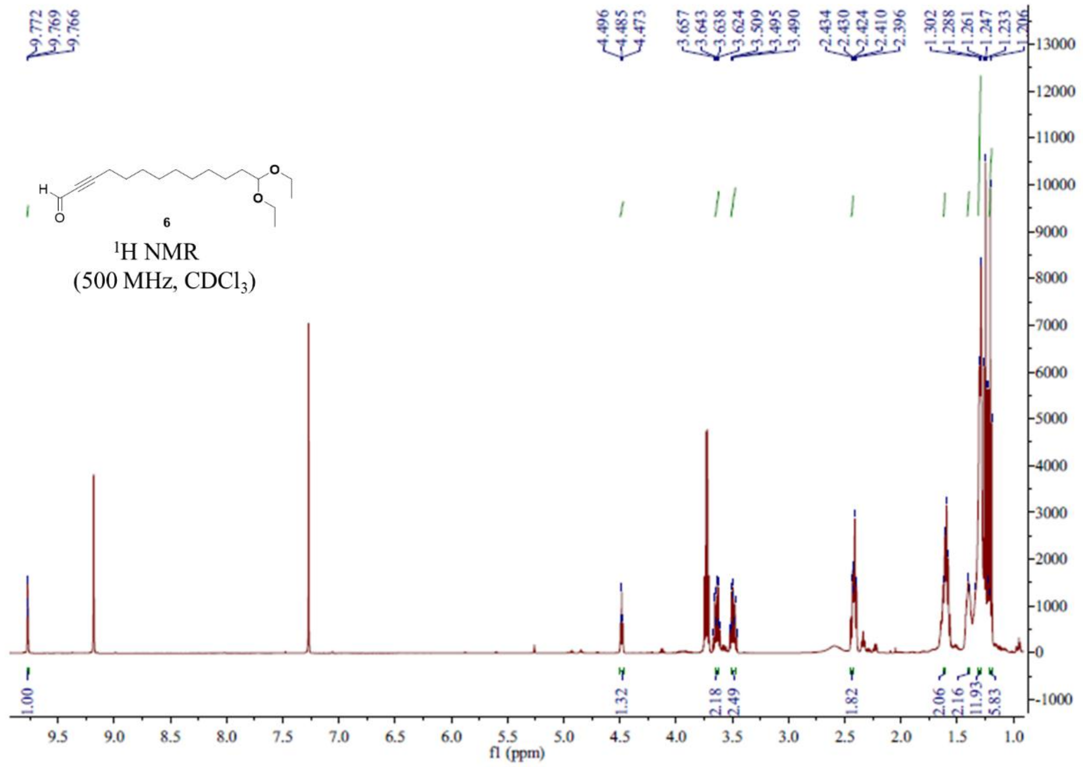

The ^{1}H NMR spectrum of compound **6**

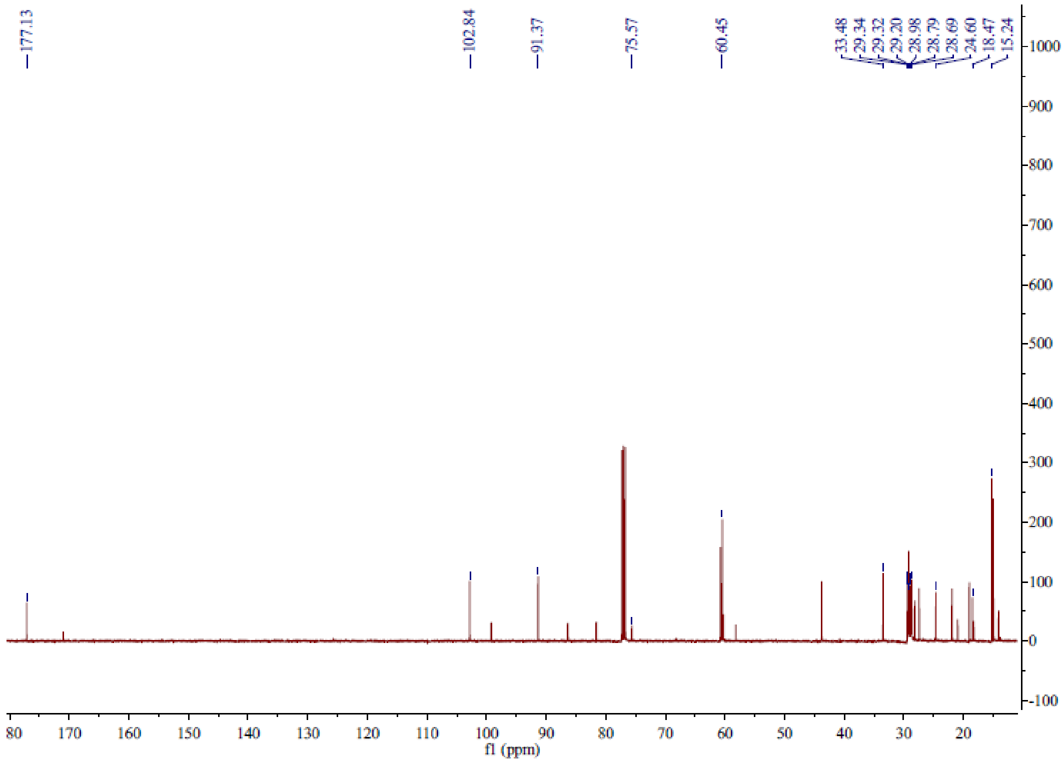

The ^{13}C NMR spectrum of compound **6**

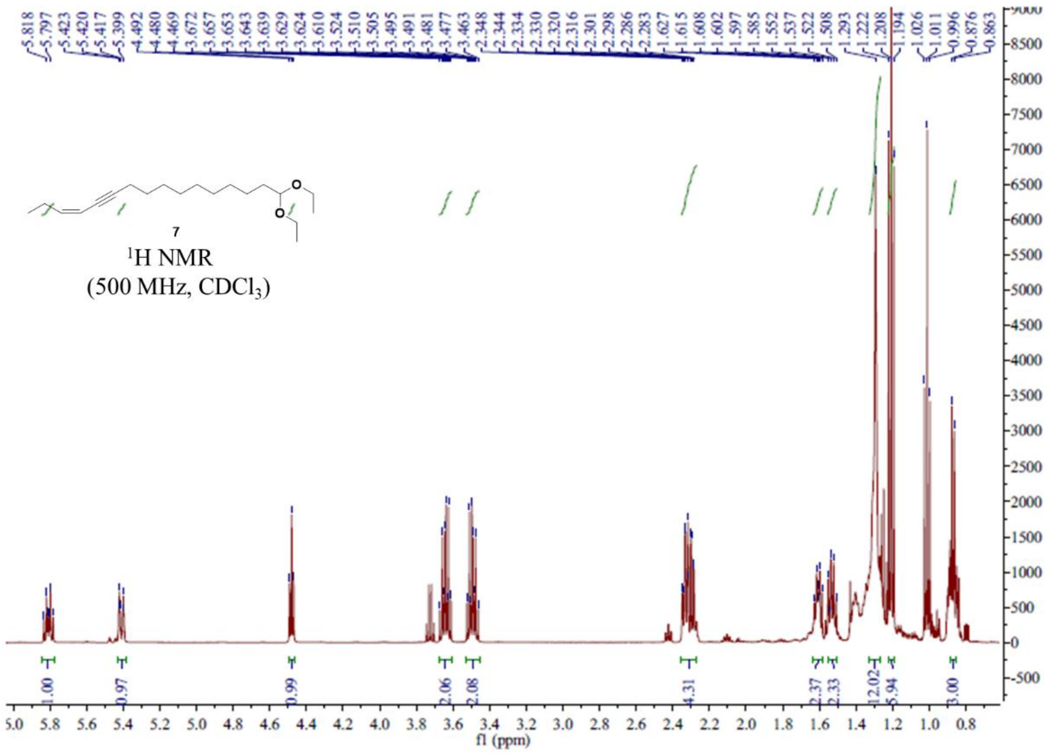

The ^{1}H NMR spectrum of compound **7**

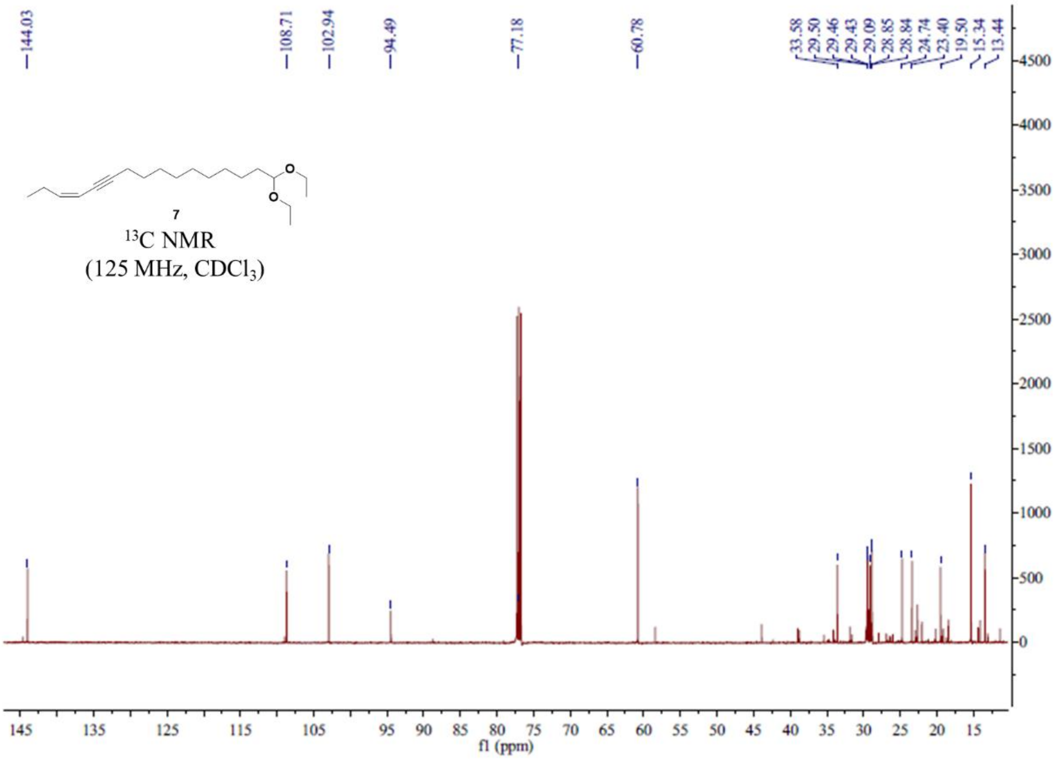

The ^{13}C NMR spectrum of compound **7**

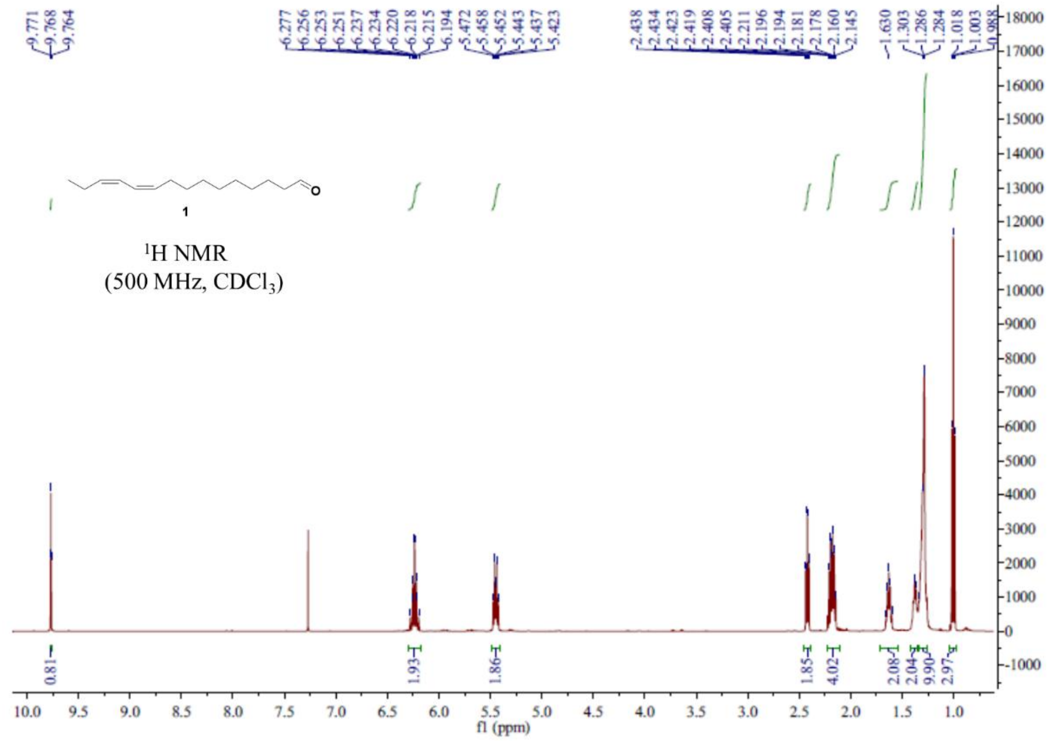

The ^{1}H NMR spectrum of compound **1**

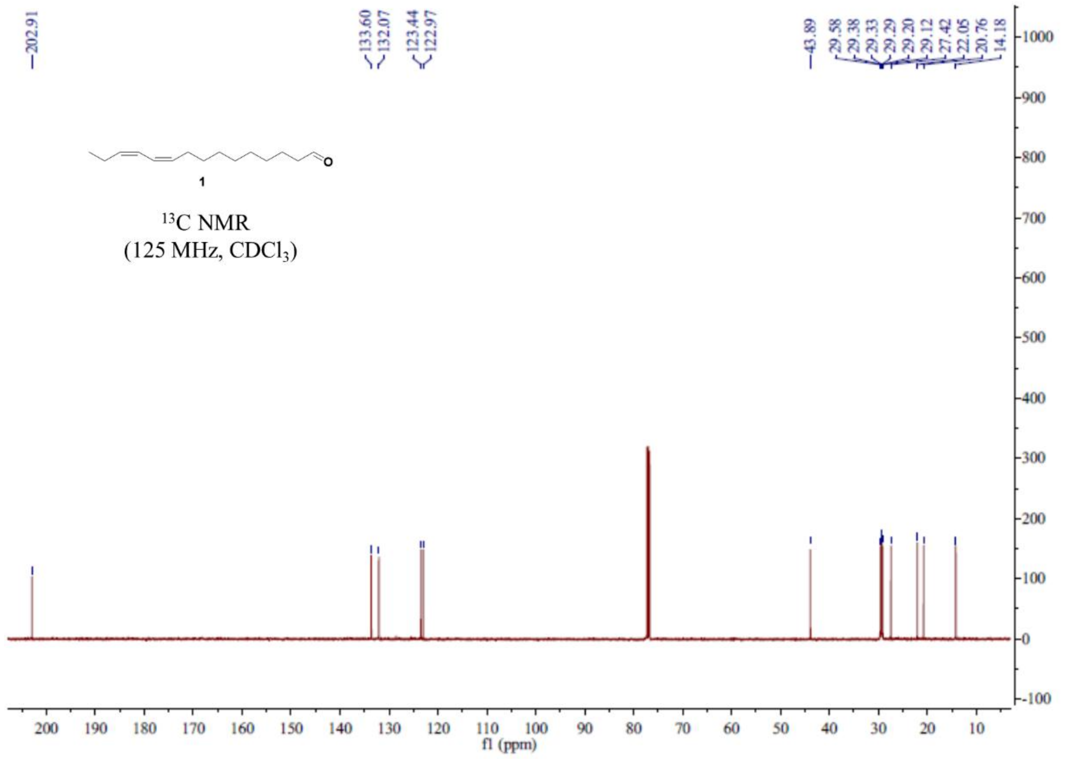

The ^{13}C NMR spectrum of compound **1**

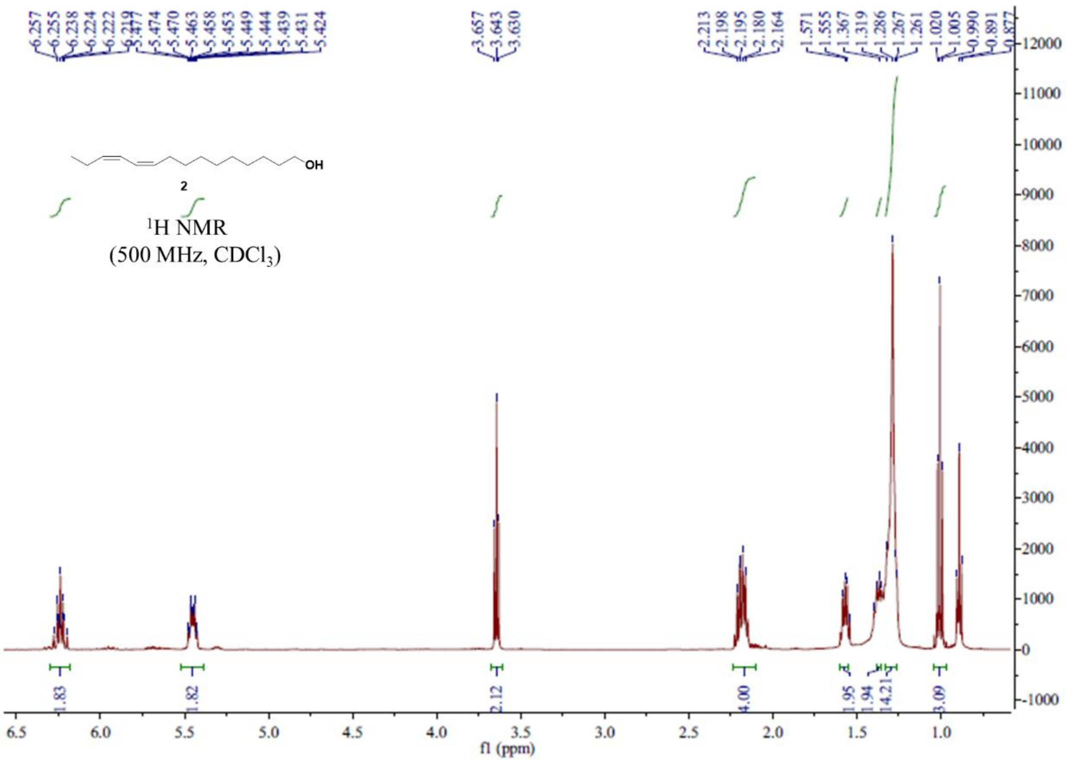

The ^{1}H NMR spectrum of compound **2**

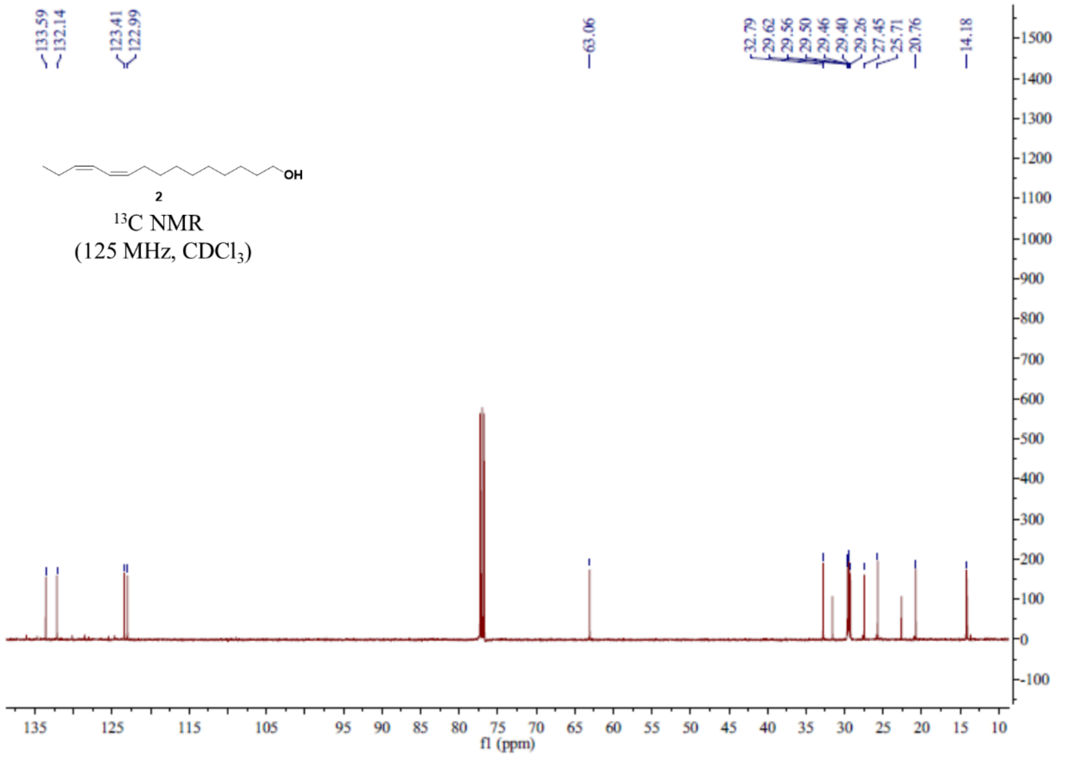

The ^{13}C NMR spectrum of compound **2**

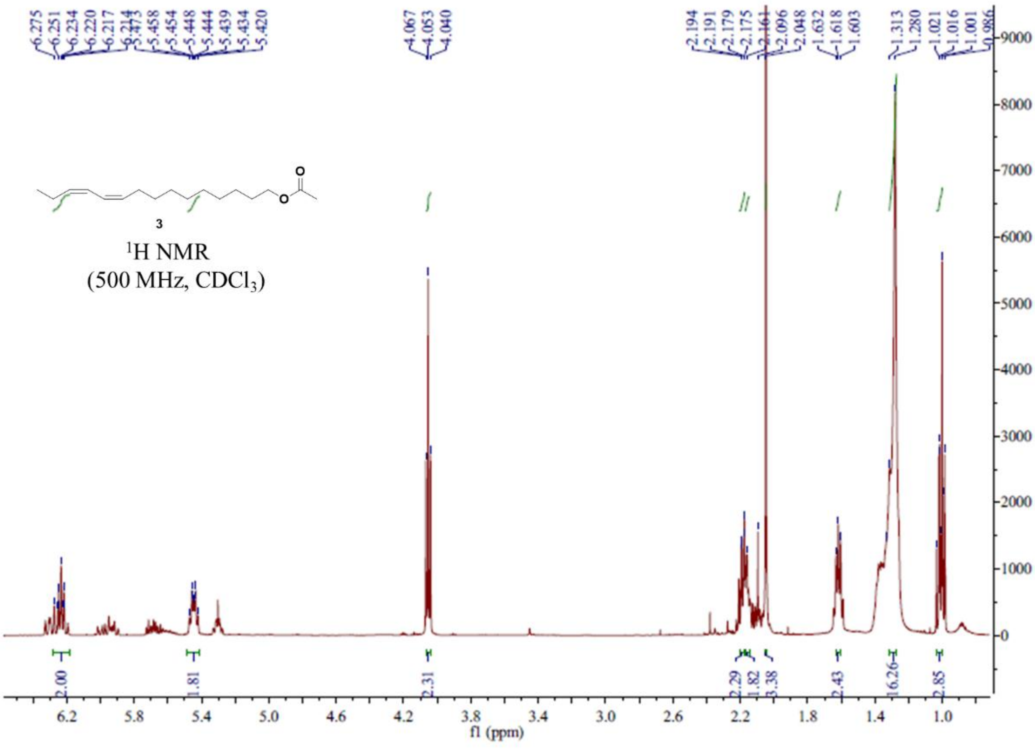

The ^{1}H NMR spectrum of compound **3**

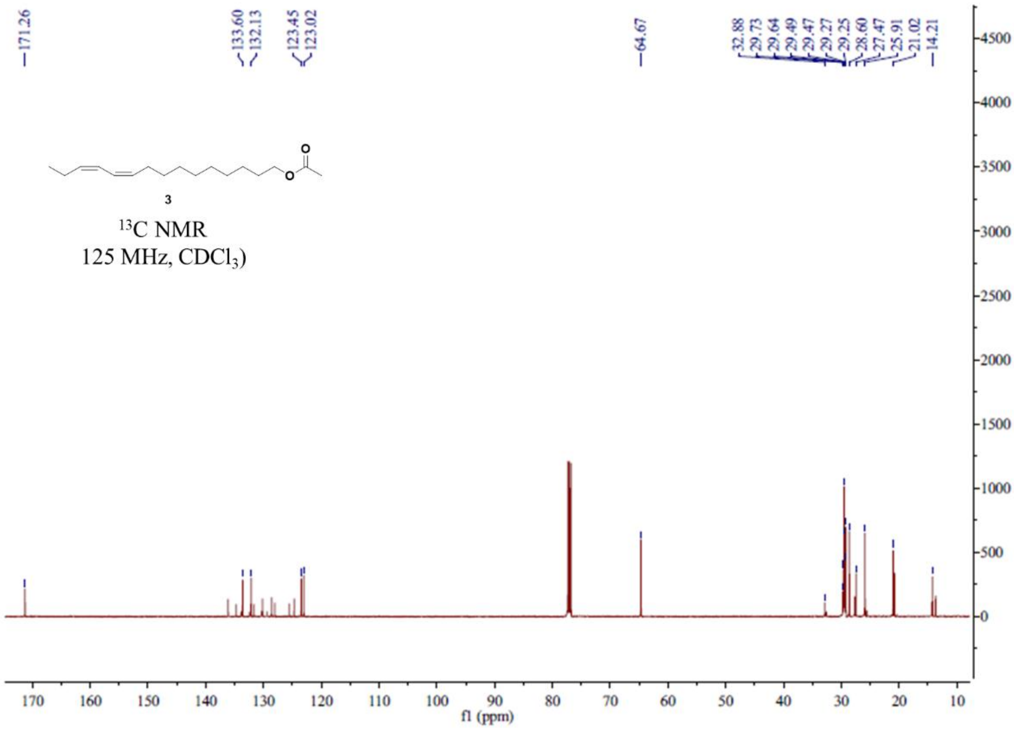

The ^{13}C NMR spectrum of compound **3**

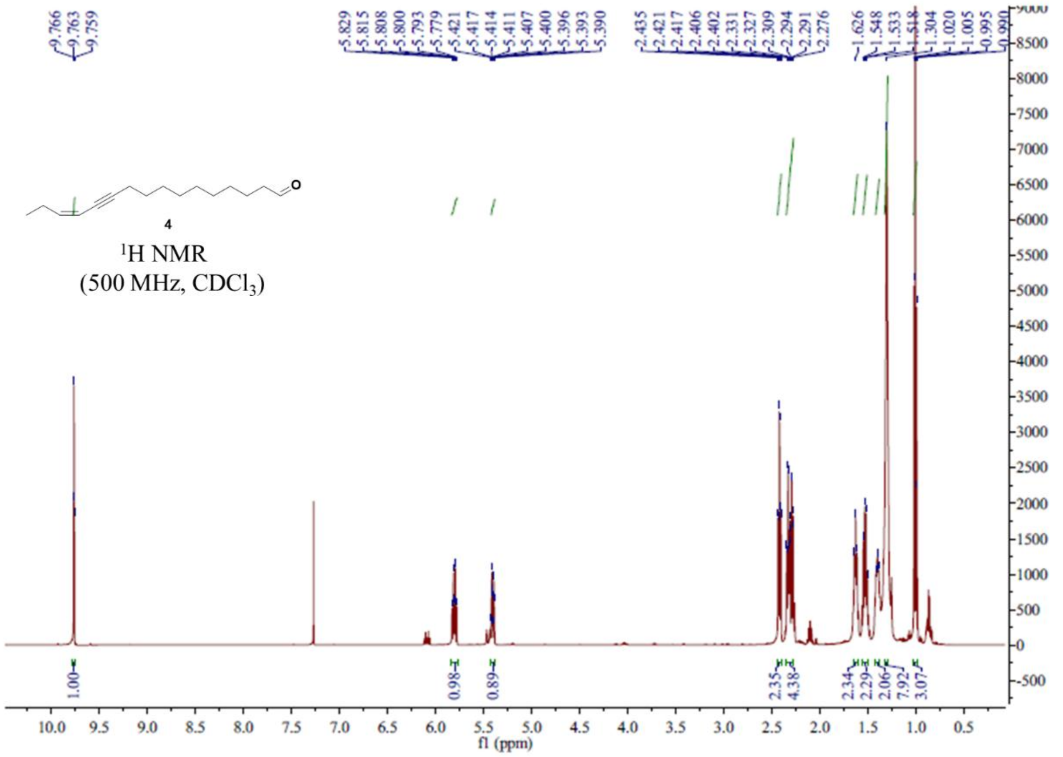

The ^{1}H NMR spectrum of compound **4**

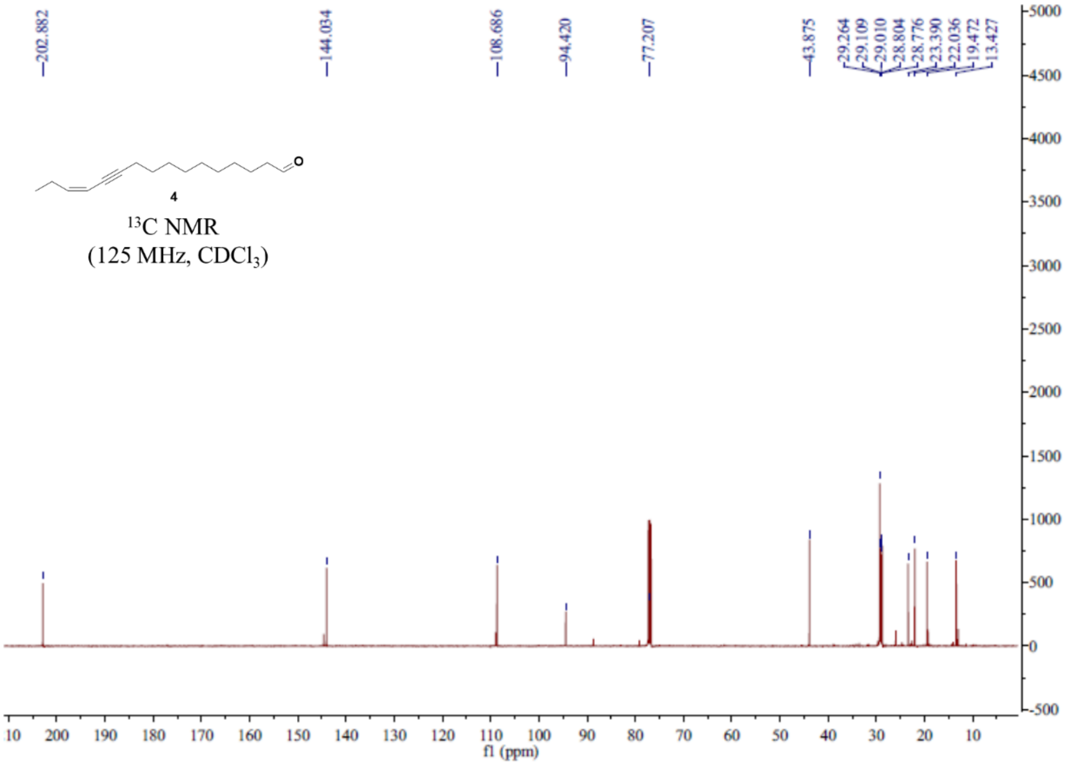

The ^{13}C NMR spectrum of compound **4**
